# The heating efficiency of magnetic nanoparticles under an alternating magnetic field

**DOI:** 10.1038/s41598-022-20558-0

**Published:** 2022-09-26

**Authors:** Xiaogang Yu, Renpeng Yang, Chengwei Wu, Bo Liu, Wei Zhang

**Affiliations:** 1 State Key Laboratory of Structure Analysis for Industrial Equipment, Department of Engineering Mechanics, Dalian University of Technology, Dalian, 116024 China; 2 School of Biomedical Engineering, Dalian University of Technology, Dalian, 116024 China

**Keywords:** Cancer, Materials science, Nanoscience and technology

## Abstract

Hysteresis loss and relaxation loss are the two dominant heating mechanisms of magnetic nanoparticles (MNPs) in an alternating magnetic field (AMF). In magnetic induction hyperthermia, heating efficiency is one of the crucial factors. It is proposed that the MNPs with a dominant heating mechanism of relaxation loss will exhibit a higher heating efficiency. However, the relative experiments supporting the proposal is still absent due to the difficulty of obtaining the MNPs with the same components and similar morphology but different dominant heating mechanism. Here, the post-processing method of calcination is employed to change the cation distribution of the MNPs (*Fe*_*3*_*O*_*4*_ and *Zn*_*0.54*_*Co*_*0.46*_*Cr*_*0.6*_*Fe*_*1.4*_*O*_*4*_), so as to obtain the MNPs with similar morphology but different dominant heating mechanism. The magnetic heating experiments were conducted to examine the heating efficiency. The results suggest that the MNPs with relaxation loss have a higher heating efficiency under the investigated AMF.

## Introduction

Magnetic induction hyperthermia (MIH) is a promising cancer therapy proposed by Gilchrist et al*.* in 1957, in which magnetic nanoparticles (MNPs) are introduced into tumor tissue to generate heat under an alternating magnetic field (AMF) for killing tumor cells^1^. The efficiency of MNPs converting electromagnetic energy to thermal energy, *i.e.*, heating efficiency, is decisive for the performance of MNPs in MIH. Hysteresis loss and relaxation loss are the two major heating mechanisms of MNPs in an AMF^2^. It is proposed that under the AMF below the biological safety limit (*H*·*f* ≤ 5 × 10^9^ Am^−1^s^−1^^3^), the MNPs relying on relaxation loss have a higher heating efficiency^4–7^. To verify this proposal, the MNPs with the same component and morphology but different dominant heating mechanisms (*i.e.*, hysteresis loss versus relaxation loss) upon AMF need to be prepared, since the heating efficiency is affected by component^8^ and morphology (shape and size)^9^ of MNPs . However, so far, it is still a challenge to obtain such types of MNPs and the experimental support for this proposal is still absent.

The dominant heating mechanism of MNPs is determined by magnetic states, *i.e.*, ferro/ferri-magnetic or superparamagnetic one^10^. Generally, the hysteresis loss is the mechanism dominantly occurs in ferro/ferr-magnetic nanoparticles, while the relaxation loss is mainly responsible for superparamagnetic nanoparticles^11–13^. However, the magnetic state of MNPs is affected by the morphology (shape and size). Generally, the particles with a size of micro/nano scale display ferro/ferri-magnetic state with a dominant heating mechanism of hysteresis loss. When the size decreases below the superparamagnetic critical size, the particles will display superparamagnetic state with a dominant heating mechanism of relaxation loss^14,15^. In other words, it is difficult for particles with the same components and similar morphology to have different dominant heating mechanism, and it is difficult to determine which heat mechanism has a higher heating efficiency by experiments. Fortunately, the magnetic state of MNPs not only depends on the morphology, but also on the cation distribution. *Tian *et al*.*^16^ showed that the cation distribution of Zn-Cr spinel nanoparticles is changed by calcination. And it was also demonstrated that the morphology of the nickel ferrite nanoparticles does not change significantly after calcination^17^. Inspired by these results, herein, we attempted to change the magnetic state of the MNPs by calcination without affecting the morphology, endowing the possibility to compare the heating efficiency of hysteresis loss with relaxation loss.

The hydrothermal method and calcination were employed to obtain the MNPs with similar morphology and different dominant heating mechanism. Then the magnetic heating experiments upon AMF were conducted to characterize the heating efficiency. The results suggested that the MNPs with a dominant heating mechanism of relaxation loss exhibit a higher heating efficiency, supporting the reported proposal experimentally.

## Material and methods

### Materials

Ferric chloride hexahydrate (FeCl_3_·6H_2_O, ≥ 99%), chromium chloride hexahydrate (CrCl_3_·6H_2_O, ≥ 99%) and zinc chloride (ZnCl_2_, ≥ 98%) were purchased from Shantou Xilong Chemical Co. Ltd., China. Cobalt chloride hexahydrate (CoCl_2_·6H_2_O, ≥ 99%) and sodium hydroxide (NaOH, ≥ 96%) were obtained from Tianjin Bodi Chemical Co. Ltd, China. All chemicals were used without further purification. The *Fe*_*3*_*O*_*4*_ nanoparticles (denoted as FO) were purchased from Aladdin Industrial Corporation, Shanghai, China.

### Synthesis of nanoparticles

The MNPs of *Zn*_0.54_*Co*_0.46_*Cr*_0.6_*Fe*_1.4_*O*_4_ were synthesized by hydrothermal method. 23.1 mmol FeCl_3_·6H_2_O, 9.9 mmol CrCl_3_·6H_2_O, 7.59 mmol CoCl_2_·6H_2_O, and 8.91 mmol ZnCl_2_ were dissolved in 80 mL deionized water to form a clear metal salts solution, and then 150 mL NaOH solution was added dropwise under magnetic stirring at room temperature. When 2 mol·L^−1^ NaOH solution was used, the resultant MNPs was denoted as ZCF-2. Whereas 3 mol·L^−1^ NaOH solution was employed, the resultant MNPs was denoted as ZCF-3. The resultant mixture was transferred to a sealed autoclave for hydrothermal treatment at 350 °C for 6 h. Then, the autoclave was cooled to room temperature naturally. After washing with deionized water and ethanol, the obtained MNPs were dried at 80 °C for 8 h in vacuum drying chamber.

### Calcination

Under the protection of nitrogen, the synthesized MNPs of ZCF-2, ZCF-3 and the purchased FO were calcined at 500 °C for 2 h in a tube furnace to form the calcined MNPs, denoted as C/ZCF-2, C/ZCF-3 and C/FO, respectively. The heating rate was set as 10 °C·min^−1^.

### Characterization

The transmission electron microscopy (TEM, FEI Tecnai G2 F30, USA) was carried out to obtain the morphology the MNPs. The X-ray powder diffractometer (XRD, PANalytical Empyrean X-ray diffractometer with Cu-Kα radiation, *λ* = 1.54056 Å, Netherlands) was utilized to determine the crystalline structure. The magnetization curve was recorded on the vibrating sample magnetometer (VSM, LakeShore 7400 s, USA) at room temperature with the applied magnetic field of ± 20 kOe.

To obtain the heating efficiency, 112 mg MNPs was dispersed in 1 mL deionized water to form magnetic suspension. After that, the MNPs suspension was placed in the AMF with a frequency of 100 kHz and the intensities of 16 kA·m^−1^ and 24 kA·m^−1^. The time-dependent temperature curves can be obtained by measuring the MNPs suspension temperature.

## Results and discussion

### Morphology and structure analysis

**Figure 1:**
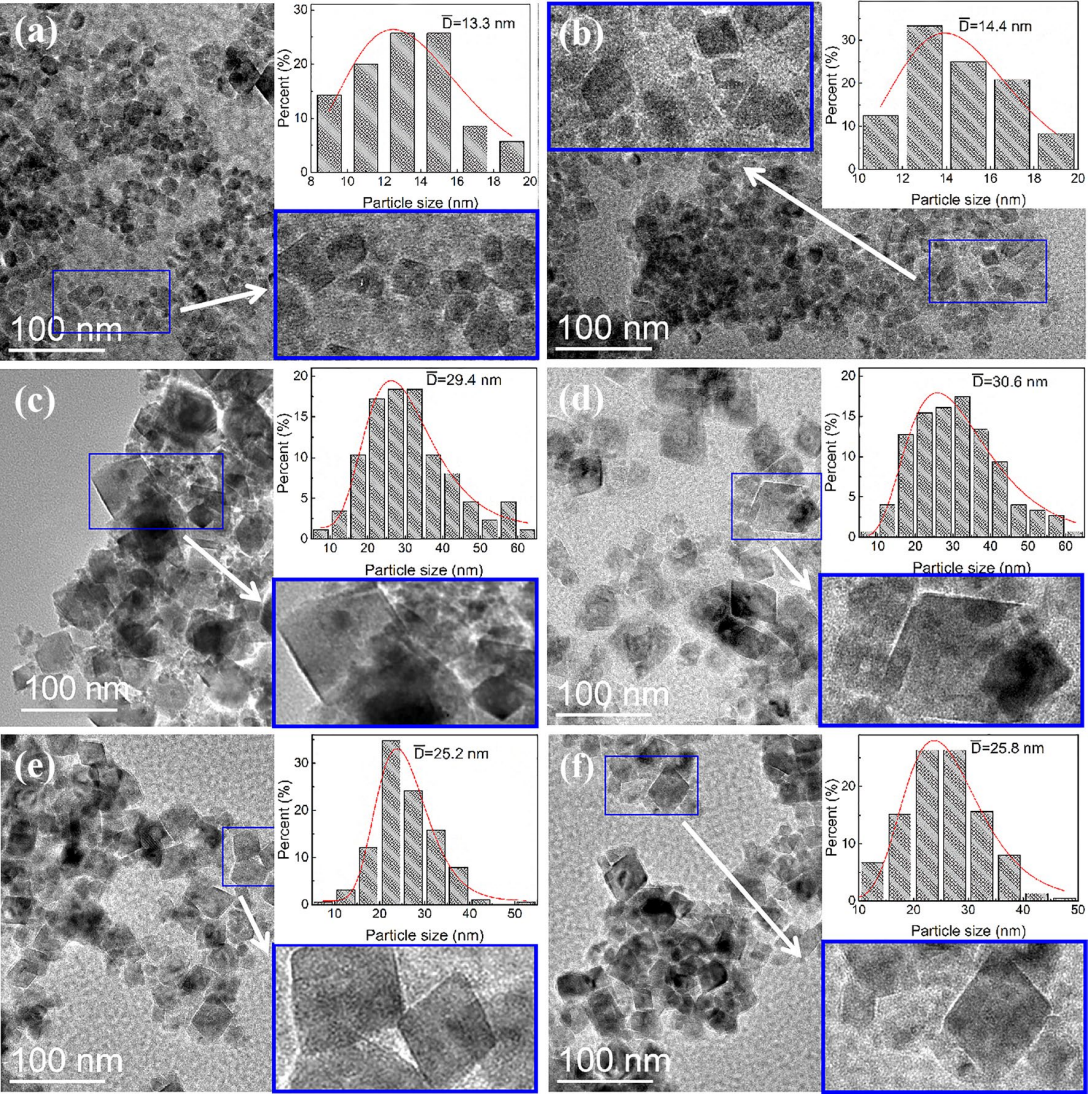
TEM images of MNPs and size distributions (the insets): (**a**) FO, (**b**) C/FO, (**c**) ZCF-3, (**d**) C/ZCF-3, (**e**) ZCF-2, (**f**) C/ZCF-2.

The morphology images of the samples obtained via the TEM are given in Fig. 1. Figure 1a, b show that FO and C/FO display imperfect tetragon with the average sizes of 13.3 nm and 14.4 nm. Figure 1c-f suggest that the morphologies of the ZCF-3, C/ZCF-3, ZCF-2 and C/ZCF-2 are regular tetragons with the average sizes of 29.6 nm, 30.6 nm, 25.2 nm and 25.8 nm, respectively. It can be seen that the shape and size of the samples after calcination are close to that before calcination. In addition, the size of ZCF-3 is larger than that of ZCF-2, which may be caused by the higher concentration of NaOH used in preparation. The lower alkali environment results in a smaller coefficient of viscosity, leading to severe Brownian movements of the grains. Thus, the crystal nucleus can form more rapidly, yielding a rapid decrease of concentration of the reacting cations and thereby a smaller crystal size^18^.

**Figure 2:**
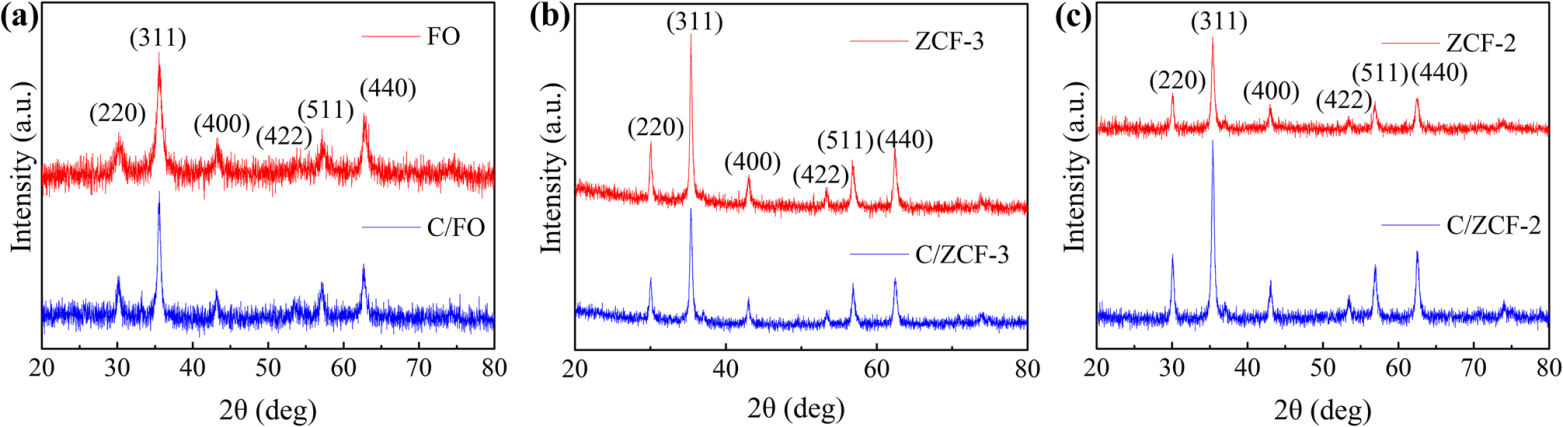
X-ray diffraction patterns of MNPs: (**a**) FO and C/FO, (**b**) ZCF-3 and C/ZCF-3, (**c**) ZCF-2 and C/ZCF-2.

Figure 2 illustrates the X-ray diffraction patterns of the samples. No obvious impurity peaks are observed except the diffraction peaks corresponding to the planes of (220), (311), (400), (422), (511) and (440), indicating the pure cubic spinel phase of these samples. The peaks of C/FO and C/ZCF-2 become sharper and narrower compared with FO and ZCF-2, suggesting that the crystallinity is increased by calcination. The same phenomenon was also observed in other studies^19,20^. In practice, the calcination can be applied to improve the crystallinity of ferrite^21^. However, the crystallinity of ZCF-3 is weakened after calcination, as the peak of ZCF-3 is sharper than that of C/ZCF-3, and the reasons need further study.

### Magnetic properties

**Figure 3:**
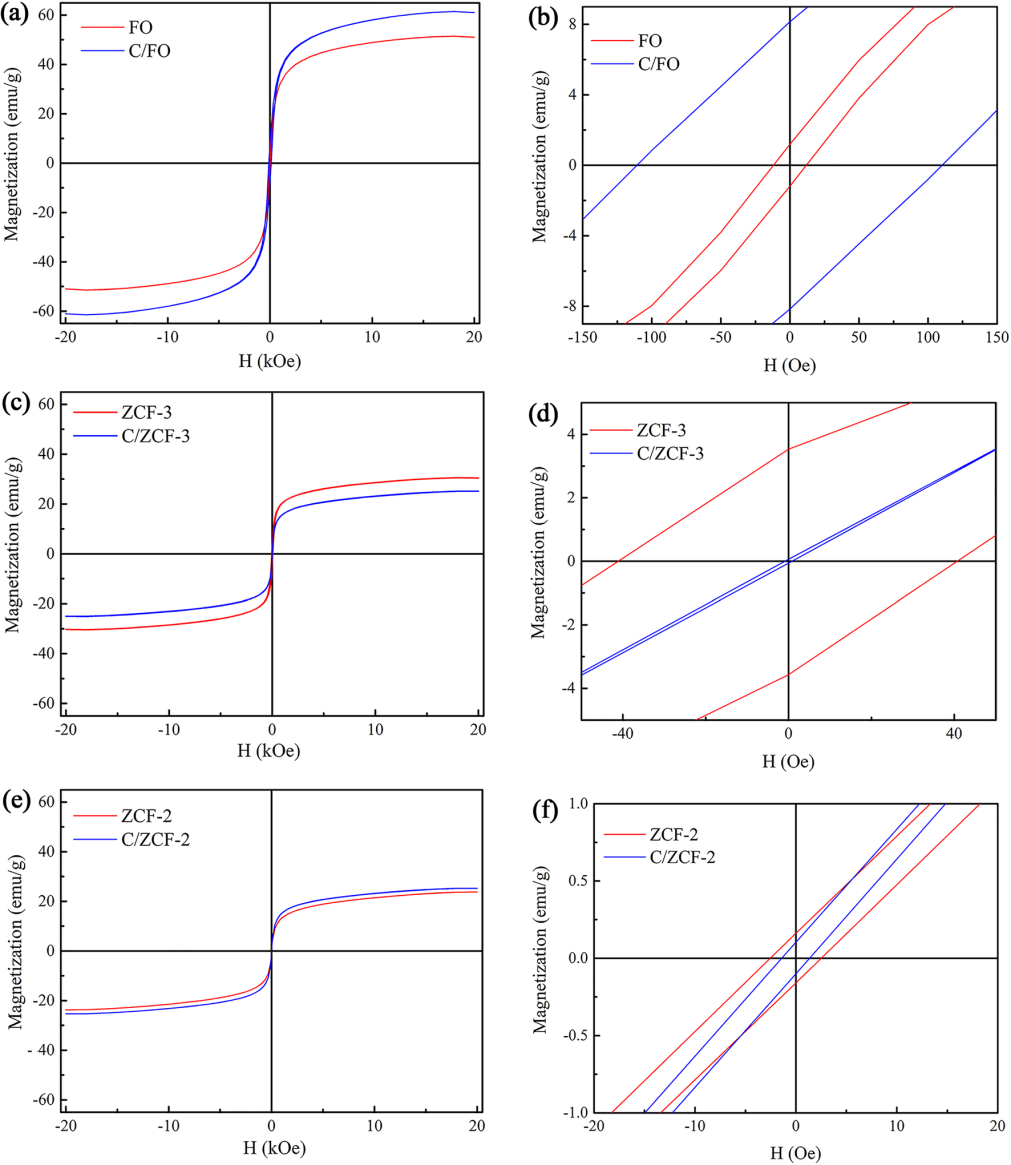
The magnetization curves (**a**, **c**, **e**) and their partial enlarged detail (**b**, **d**, **f**) of the samples: (**a**, **b**) FO and C/FO, (**c**, **d**) ZCF-3 and C/ZCF-3, (**e**, **f**) ZCF-2 and C/ZCF-2.

The magnetization curves measured at room temperature via VSM are shown in Fig. 3. It shows that the specific saturation magnetization (*σ*_*s*_) of FO is 51.5 emu·g^−1^, much larger than that of ZCF-3 and ZCF-2, which are 30.5 emu·g^−1^ and 23.7 emu·g^−1^ respectively. This is mainly attributed to the higher content of Zn^2+^ in ZCF-3 and ZCF-2. Because of the strong A sites occupation tendency of the nonmagnetic Zn^2+^, the doped Zn^2+^ will mainly occupy the A sites^22^. When the doped content of Zn^2+^ is much larger, the magnetic moments of A sites will decrease so much that the dominant super-exchange interaction of A-B becomes much weaker and the B-B super-exchange interaction strengthens, which leads to the occurrence of random spin canting on the B sites with respect to the direction of spins of the A sites, resulting in the decrease of the specific saturation magnetization^23^. In addition, the *σ*_*s*_ of ZCF-3 is larger than that of ZCF-2, which can be ascribed to the larger size of ZCF-3, as seen Fig. 1c, e.

Figure 3a, b show the magnetization curves and the partial enlarged details of FO and C/FO. It can be seen that FO has a specific saturation magnetization (*σ*_*s*_) of 51.5 emu·g^−1^ with a coercivity (*H*_*c*_) of 11.9 Oe, indicating the superparamagnetic state of FO, since the *H*_*c*_ is less than one thousandth of the required intensity to reach 90% of the specific saturation magnetization^24^. After calcination, C/FO displays a ferrimagnetic state, of which *σ*_*s*_ and *H*_*c*_ are 61.5 emu·g^−1^ and 110.5 Oe, respectively. Figure 3c, d exhibit the magnetization curves and the partial enlarged detail of ZCF-3 and C/ZCF-3. It can be seen that the *σ*_*s*_ decreases from 30.5 emu·g^−1^ to 25.1 emu·g^−1^ and the *H*_*c*_ decreases from 40.9 Oe to 1.0 Oe, suggesting that the calcination transforms the ferrimagnetic ZCF-3 to superparamagnetic C/ZCF-3. The magnetization curves and the partial enlarged detail of ZCF-2 and C/ZCF-2 with tiny coercivities are given in Fig. 3e, f, revealing their superparamagnetic natures. In addition, the *σ*_*s*_ of C/ZCF-2 has a slight increase compared with ZCF-2, from 23.7 emu·g^−1^ to 25.3 emu·g^−1^.

The change of *σ*_*s*_ after calcination may be attributed to the change of the crystallinity, as seen in Fig. 2. The MNPs with higher crystallinity will have a thinner surface layer, which probably contributes to weaker cation disorder, resulting in a higher specific saturation magnetization^25^. As a result, the *σ*_*s*_ of C/FO and C/ZCF-2 increase due to the higher crystallinity. On the contrary, due to the reduction of crystallinity, C/ZCF-3 has a smaller value of *σ*_*s*_.

The change of coercivity after calcination may be ascribed to the change of cation distribution. It is known that the coercivity of MNPs is related to the particle shape, size, component, and cation distribution^26,27^. As seen in Fig. 1, the calcination does not change the component, shape and size significantly. Thus, it supposes that the change of cation distribution should be the main reason of the change of coercivity. It has been demonstrated that calcination may make the Fe^3+^ migrate from B sites to A sites, resulting an increase of coercivity^28^, which is likely to be the main reason of the increase of coercivity of C/FO. The coercivity decrease of C/ZCF-3 may be caused by the migration of Co^2+^ from B sites to A sites. It is known that the magnetocrystalline anisotropy of cobalt ferrite is mainly ascribed to the incompletely quenched orbital momentum of Co^2+^ at B sites^29^, so the migration of Co^2+^ from B sites to A sites will incur the decrease of magnetocrystalline anisotropy and the decrease of coercivity. The migration of Co^2+^ from B sites to A sites caused by calcination is reported in literature^30^. Wang et al*.*^31^ also suggested that the calcination will cause the Co^2+^ migration from B sites to A sites, resulting in the decreased coercivity. Based on the similar situation of C/ZCF-3, the coercivity of the calcinated C/ZCF-2 does no change significantly due to the tiny coercivity of the original ZCF-2.

### The heating efficiency

**Figure 4:**
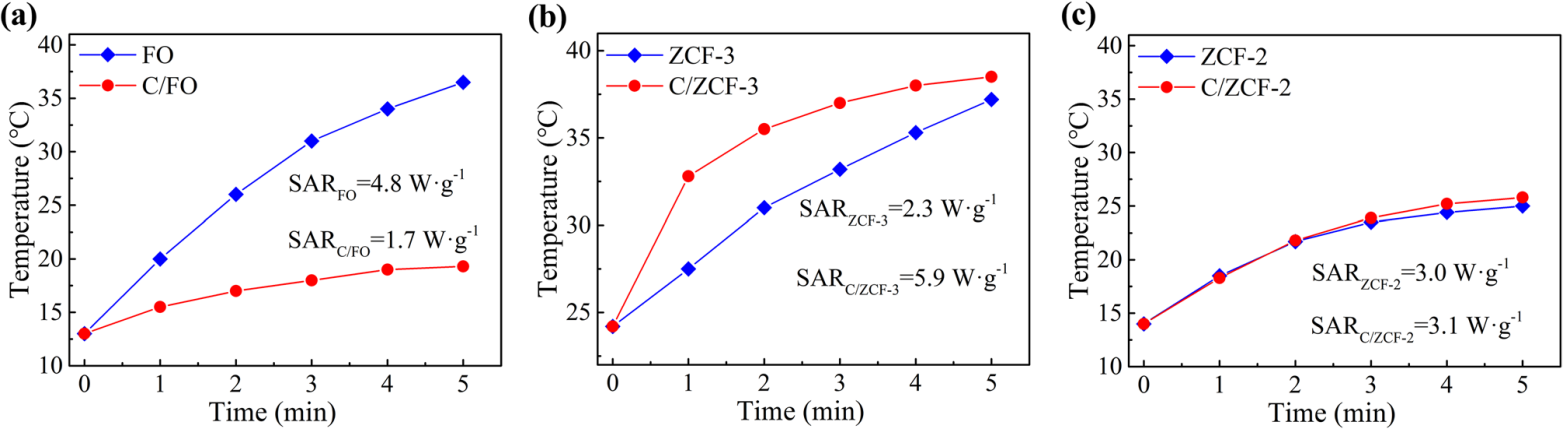
The magnetic heating curves of the samples under the AMF with 16 kA/m and 100 kHz: (**a**) FO and C/FO, (**b**) ZCF-3 and C/ZCF-3, (**c**) ZCF-2 and C/ZCF-2.

To characterize the heating efficiency, the magnetic heating experiments were conducted under an AMF with an intensity of 16 kA·m^−1^ and a frequency of 100 kHz. Once placed in the AMF, the samples convert the electromagnetic energy into thermal energy via the mechanisms of hysteresis loss or relaxation loss, and the temperatures of the MNPs suspensions begin to rise, as seen in Fig. 4. The heating efficiency of MNPs, usually expressed as the specific absorption rate (SAR), is calculated by the formula of $SAR = \left. {C\frac{{m_{s} }}{{m_{p} }}\frac{dT}{{dt}}} \right|_{t \to 0}$, where *C* is the specific heat capacity of water, *m*_*s*_ is the mass of the MNPs suspension, *m*_*p*_ is the mass of MNPs in the magnetic suspension, dT/dt|_*t*→0_ is the initial slope of the time-dependent temperature curve^32^.

According to the experimental data in Fig. 4a, the SAR of FO and C/FO are 4.8 W·g^−1^ and 1.7 W·g^−1^, respectively. As seen in Fig. 1a, b, the morphology of the samples after calcination are close to that of before calcination, which is unlikely to incur the significant change of heating efficiency. Although the *σ*_*s*_ increases after calcination, it can not be the reason of the decrease of the heating efficiency, since the MNPs with higher *σ*_*s*_ will lead to a higher heating efficiency^33,34^. As described above, compared with FO, the main change of C/FO is the change of magnetic state, *i.e.*, superparamagnetic FO is transformed into ferrimagnetic C/FO (as seen in Fig. 3a). It is known that the hysteresis loss is dominant in ferrimagnetic nanoparticles, while the relaxation loss prevails in superparamagnetic nanoparticles^11–15^. Thus, it may be inferred that the decrease of the heating efficiency of C/FO is mainly caused by the change of heating mechanism from relaxation loss to hysteresis loss, which seems to be consistent with the proposal that the relaxation loss may exhibit a higher heating efficiency than hysteresis loss. In order to further investigate this issue, the magnetic heating experiments of ZCF-3, C/ZCF-3, ZCF-2 and C/ZCF-2 were conducted and the results are shown in Fig. 4b and Fig. 4c.

After calcination, the morphology is not changed significantly (as seen in Fig. 1c, d), while the dominant heating mechanism is changed from the hysteresis loss of ZCF-3 to the relaxation loss of C/ZCF-3, since the ferrimagnetic ZCF-3 is transformed into superparamagnetic C/ZCF-3 (as seen in Fig. 3b). Thus, though the *σ*_*s*_ has a slight decrease, the heating efficiency has a significant increase from 2.3 W·g^−1^ to 5.9 W·g^−1^, as seen in Fig. 4b. As expected, the SAR of ZCF-2 and C/ZCF-2 are not obviously changed (3.0 W·g^−1^ for ZCF-2 and 3.1 W·g^−1^ for C/ZCF-2) as illustrated in Fig. 4c, since the dominant heating mechanism (relaxation loss) is not changed and the change of both morphology and *σ*_*s*_ are not pronounced (as seen in Fig. 1e, f and Fig. 3c). To sum up, the conducted magnetic heating experiments suggest that the MNPs of *Fe*_3_*O*_4_ and *Zn*_0.54_*Co*_0.46_*Cr*_0.6_*Fe*_1.4_*O*_4_ with relaxation loss have a higher heating efficiency than hysteresis loss under the AMF with 16 kA/m and 100 kHz. It needs to point out that the relaxation loss commonly includes Neel relaxation and Brownian relaxation. Aside from the intensity and frequency of the applied AMF, relaxation loss depends on the relaxation time. The Neel relaxation time depends on the magnetic state and Brownian relaxation time depends on the solvent and MNPs size. Since the sizes of the MNPs before and after calcination are similar, and the solvent used here is the same, the effect of Brownian relaxation to the variation of heating efficiency is not expected to be significant. Thus, the higher heating efficiency may be attributed to Neel relaxation loss. It is known that Neel relaxation mainly occurs in superparamagnetic nanoparticles, and this may be one of the reasons that the superparamagnetic nanoparticles has been the backbone in the development of MIH.

**Figure 5:**
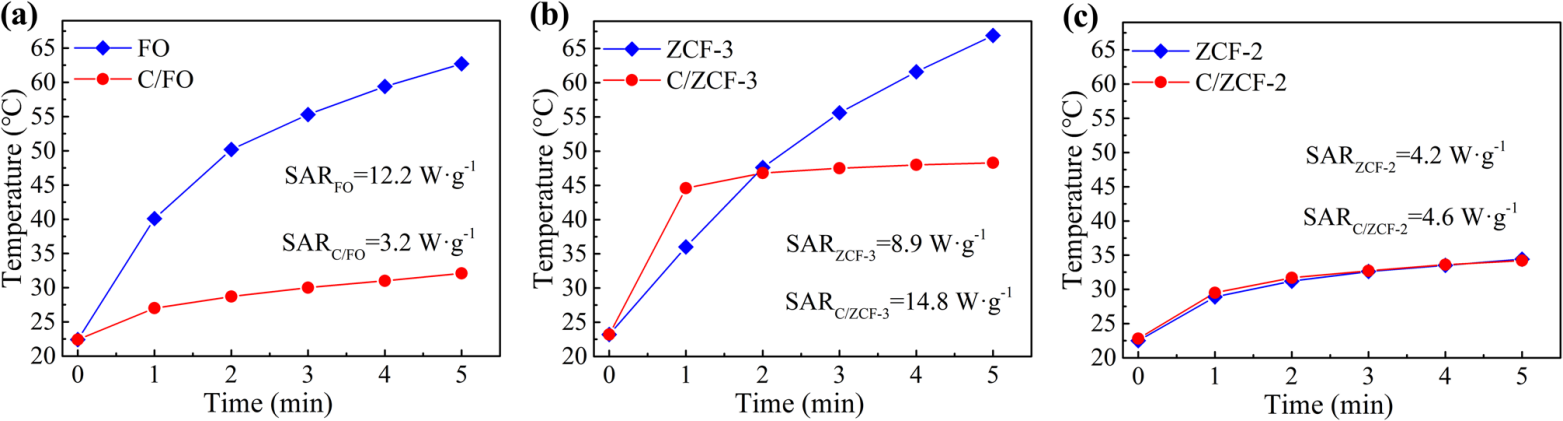
The magnetic heating curves of the samples under the AMF with 24 kA/m and 100 kHz: (**a**) FO and C/FO, (**b**) ZCF-3 and C/ZCF-3, (**c**) ZCF-2 and C/ZCF-2.

**Figure 6:**
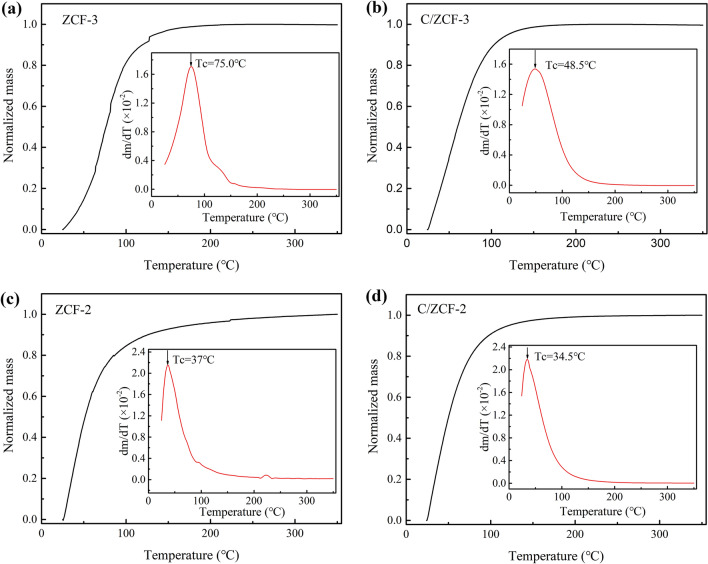
The thermogravimetric curves of MNPs under a static magnetic field: (**a**) ZCF-3, (**b**) C/ZCF-3, (**c**) ZCF-2, (**d**) C/ZCF-2. The insets are the first derivative of the corresponding thermogravimetric curves, where the temperature corresponding to the maximum value is defined as the Curie temperature.

In order to check the effect of the intensity of AMF, the heating experiments at AMF of 24 kA/m and 100 kHz were conducted, as show in Fig. 5. The experimental results suggest that the heating efficiency of *Fe*_3_*O*_4_ and *Zn*_0.54_*Co*_0.46_*Cr*_0.6_*Fe*_1.4_*O*_4_ MNPs with prevailing relaxation loss is higher than that with hysteresis loss, which is consistent with the results of heating experiments under the AMF of 16 kA/m and 100 kHz. It is worth noting that compared with ZCF-3, the C/ZCF-3 possesses a larger SAR but a lower eventual temperature, as seen in Fig. 5b. This is because that the C/ZCF-3 have a lower Curie temperature (*Tc,* as seen Fig. 6a, b), which can be ascribed to the migration of Co^2+^ ions from B sites to A sites^35^. Since the super-exchange interaction between Co^2+^ in A sites and Fe^3+^ in B sites is weaker than that between Fe^3+^ in both A sites and B sites, the Co^2+^ migration from B sites to A sites will cause the decrease of the super-exchange interaction, leading to the decrease of *Tc*^36^. Curie temperature is a phase transformation temperature of MNPs, above which the magnetic phase of MNPs will transform from ferromagentic phase to paramagnetic phase, *i.e.*, the magnetization will decrease to near zero, decreasing the heating efficiency to approximately zero. After two minutes of heating, the temperature of C/ZCF-3 suspension reaches 46.8 °C. Since this is close to the *Tc* of 48.5 °C (as seen Fig. 6b), the temperature of C/ZCF-3 suspension will level off (as seen Fig. 5b). While after two minutes of heating, the temperature of ZCF-3 suspension reaches 47.6 °C, much lower than the *Tc* of 75.0 °C (as seen Fig. 6a). The temperature of ZCF-3 suspension will continue to rise beyond that of C/ZCF-3 suspension (as seen Fig. 5b). Similarly, due to the effect of low *Tc*, the temperature of ZCF-2 and C/ZCF-2 suspensions rise to only 34.4 °C and 34.2 °C (as seen Fig. 5c). The *Tc* of ZCF-2 and C/ZCF-2 are shown in Fig. 6c, d. The *Tc* of ZCF-2 and C/ZCF-2 are lower than that of C/ZCF-3, which may be ascribed to their smaller sizes (as seen Fig. 1d-f). Compared with the significant change of *Tc* of ZCF-3 after calcination (from 75.0 °C to 48.5 °C), the calcination only incurs a slight decrease in *Tc* of ZCF-2, *i.e.*, from 37.0 °C to 34.5 °C. This may be ascribed to the fact that the Co^2+^ occupation rate at B sites in ZCF-2 is not high, as evidenced by the small coercivity of ZCF-2, as seen Fig. 3c. As such, the migration of Co^2+^ from B sites to A sites is not expected to be significant. In brief, the magnetic heating experiments under different intensities of AMF support the proposal in the literature^4–7^ that the MNPs relying on relaxation loss have a higher heating efficiency under the AMF.

## Conclusion

In this paper, the MNPs with the same component and similar morphology but different dominant heating mechanism are obtained by the hydrothermal method and calcination. The magnetization curves show that the magnetic state of *Fe*_*3*_*O*_*4*_ is transformed from superparamagnetic (labeled as FO) to ferrimagnetic (labeled as C/FO) after calcination, suggesting the dominant heating mechanism is changed from relaxation loss to hysteresis loss. Additionally, comparing with superparamagnetic FO, the specific saturation magnetization of ferrimagnetic C/FO increases. The results of magnetic heating experiments suggest that the heating efficiency of FO is higher than that of C/FO, which supports the proposal that the relaxation loss may exhibit a higher heating efficiency than hysteresis loss. The heating experiments of *Zn*_0.54_*Co*_0.46_*Cr*_0.6_*Fe*_1.4_*O*_4_ were conducted to characterize the heating efficiency for better clarifying the problem. The magnetic state of *Zn*_0.54_*Co*_0.46_*Cr*_0.6_*Fe*_1.4_*O*_4_ is changed from ferrimagnetic (ZCF-3) to superparamagnetic (C/ZCF-3) after calcination and the heating efficiency is increased as expected. The magnetic states of *Zn*_0.54_*Co*_0.46_*Cr*_0.6_*Fe*_1.4_*O*_4_ before (ZCF-2) and after (C/ZCF-2) calcination are all superparamagnetic, and their heating efficiencies are fairly close to each other. In summary, this paper provides an experiment support for the proposal that the MNPs relying on relaxation loss have a higher heating efficiency upon AMF below the biological safety limitation.

## Supplementary Information

Supplementary Information.

## Data Availability

The datasets used and/or analysed during the current study are availability from the corresponding author on reasonable request.
