# Supplementary material for: The heating efficiency of magnetic nanoparticles under an alternating magnetic field
Source: Sci Rep. 2022 Sep 26;12:16055. doi: 10.1038/s41598-022-20558-0 (PMC9513098; doi:10.1038/s41598-022-20558-0)
Supplement: Supplementary file 1 — Supplementary Information. [file 41598_2022_20558_MOESM1_ESM.docx]

**Supporting information**

**The heating efficiency of magnetic nanoparticles under an alternating magnetic field**

Xiaogang Yu ^a, b^, Renpeng Yang ^a^, Chengwei Wu ^a^, Bo Liu ^b^, Wei Zhang ^a,^ ^[[1]](#footnote-0)^*

^a^ State Key Laboratory of Structure Analysis for Industrial Equipment, Department of Engineering Mechanics, Dalian University of Technology, Dalian 116024, China

^b^ School of Biomedical Engineering, Dalian University of Technology, Dalian 116024, China

**1 Morphology and magnetic properties**

Fig.S1 exhibits the morphology images and size distributions of *Zn*_0.54_*Co*_0.46_*Cr*_0.6_*Fe*_1.4_*O*_4_ MNPs synthesized with different NaOH concentrations. Fig.S1(a)-(c) are corresponding to the MNPs with NaOH concentrations of 5 mol/L, 6 mol/L and 7 mol/L, respectively. And the average size of MNPs against with NaOH concentration is given in Fig.S1(d). In can be seen that the average size of MNPs increases with the NaOH concentration. It is because that the lower alkali environment results in a smaller coefficient of viscosity, leading to severe Brownian movements of the grains. Thus, the crystal nucleus can be formed more rapidly, yielding a rapid decrease of the reacting cations and thereby a smaller crystal size [1]. Fig.S2 is the magnetization curves of *Zn*_0.54_*Co*_0.46_*Cr*_0.6_*Fe*_1.4_*O*_4_ MNPs synthesized with various NaOH concentrations and the paritial enlarged detail. It can be seen from Fig.S2(a) that the specific saturation magnetization increases with the NaOH concentration, which is attributed to the increase on size. Since the magnetization is the result from the ordered arrangement of magnetic moments, the decrease in MNPs size will incur high surface curvature, which in turn leads to an increase in magnetic disorder, resulting in a decrease in specific saturation magnetization [2]. Fig.S2(b) shows that the coercivity increase with the NaOH concentration, which is also attributed to the increase on size. Commonly, the coercivity varies with the size of MNPs. When the size is below the critical size for superparamagnetic, the coercivity of MNPs is negligible. After that, the coercivity will increase with the size until the size reaches the critical single-domain size, where the coercivity reaches the maximum value [3].

**
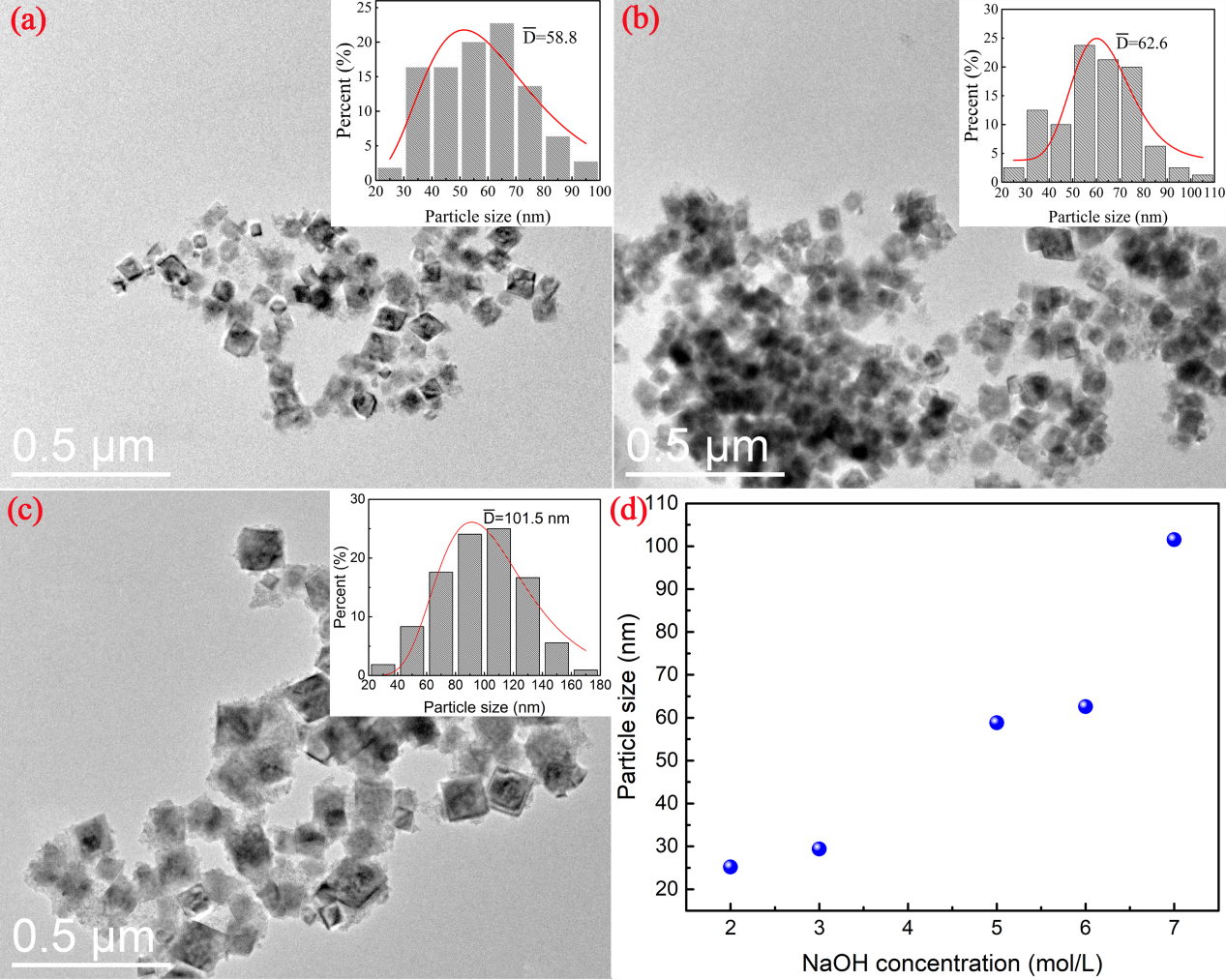
**

Fig. S1 TEM images and size distributions (the insets) of *Zn*_0.54_*Co*_0.46_*Cr*_0.6_*Fe*_1.4_*O*_4_ MNPs synthesized with different NaOH concentrations: (a) 5 mol/L, (b) 6 mol/L, (c) 7 mol/L; (d) the change of size against the NaOH concentration (the size distributions of MNPs corresponding to the NaOH concentrations of 2 and 3 mol/L are shown in the manuscript).

**
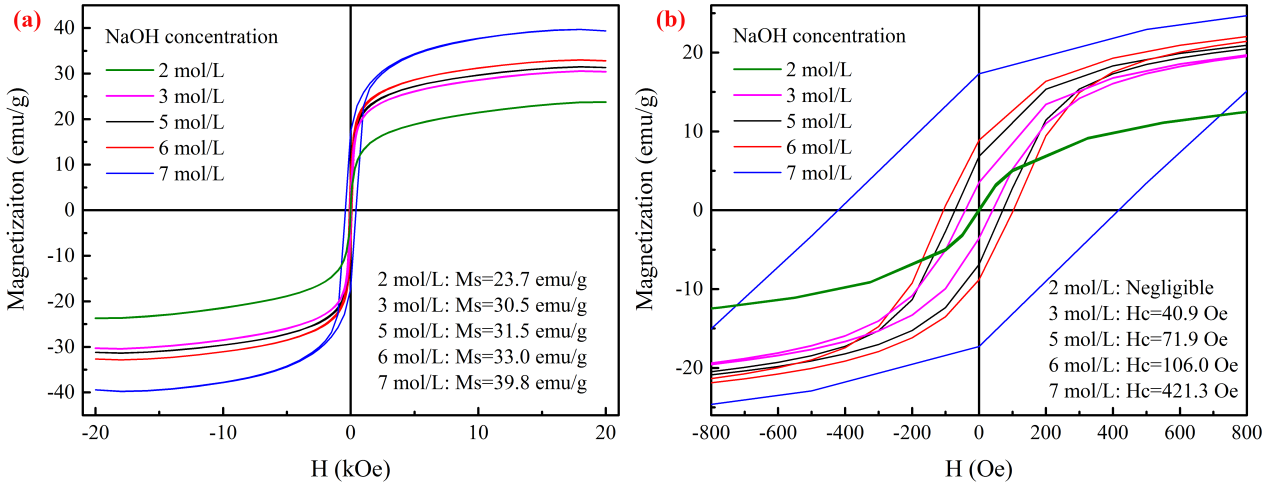
**

Fig. S2 The magnetization curves of *Zn*_0.54_*Co*_0.46_*Cr*_0.6_*Fe*_1.4_*O*_4_ MNPs synthesized with various NaOH concentrations (a), the partial enlarged detail (b).

**References**

1. Lv, L., *et al*. Grain size effect on the dielectric and magnetic properties of NiFe2O4 ceramics. *Physica E* **43**, 1798-1803 (2011).
2. Sun, C., Lee, J. S. H., Zhang, M. Magnetic nanoparticles in MR imaging and drug delivery. *Adv. Drug Deliver. Rev.* **60**, 1252-1265 (2008).
3. Shaterabadi, Z.,Nabiyouni, G., Soleymani, M. Physics responsible for heating efficiency and self-controlled temperature rise of magnetic nanoparticles in magnetic hyperthermia therapy. *Prog. Biophys. Mol. Biol.* **133**, 9-19 (2018).

1. * Corresponding authors. Tel: +86 411 84706353, E-mails: [wei.zhang@dlut.edu.cn](mailto:wei.zhang@dlut.edu.cn) [↑](#footnote-ref-0)
